# Strangulated Diaphragmatic Hernia Following Roux-en-Y Gastric Bypass: Surgical Pitfalls and Lessons Learned

**DOI:** 10.1007/s11695-025-08303-8

**Published:** 2025-10-27

**Authors:** Ahmed Abdelsalam, Mohammed Elshal, Ahmed Elansary, Ahmed Khaled

**Affiliations:** Cairo University, Giza, Egypt

**Keywords:** Iatrogenic Diaphragmatic Hernias, Bariatric Surgery, RYGB Complications

## Abstract

**Introduction:**

With the rising number of MBS and their revisions, rare complications may start to increase as well. Iatrogenic diaphragmatic hernia following metabolic and bariatric surgery (MBS) is extremely rare but potentially fatal. Its recognition is often delayed due to vague clinical presentations.

**Purpose:**

We report a case of strangulated diaphragmatic hernia after Roux-en-Y gastric bypass (RYGB) and gastro-gastric fistula dismantling, highlighting diagnostic and surgical pitfalls, and lessons for bariatric surgeons.

**Methods:**

We describe the presentation, diagnostic work-up, and surgical management of a diaphragmatic hernia after RYGB.

**Results:**

The patient had a smooth postoperative recovery but then developed pleural effusion requiring tapping. She had recovered fully with normal follow-up clinically and radiologically.

**Conclusion:**

This case highlights the importance of attention to such rare but life-threatening consequences after MBS. Precise use of energy devices intraoperatively, especially in revisional cases, is mandatory. A high index of suspicion for vague abdominal symptoms and tailored surgical strategies are essential.

**Supplementary Information:**

The online version contains supplementary material available at 10.1007/s11695-025-08303-8.

## Introduction

Bariatric surgeries are now regarded as the primary sustainable management option for patients with obesity when medical interventions fail, and they are widely performed nowadays [1]. With the rising number of gastric bypass surgeries, rare complications will start to come to the surface, like diaphragmatic hernias, for instance.

Acquired diaphragmatic hernias usually result from trauma, such as blunt or penetrating injuries to the abdomen or chest. However, they can rarely be caused by surgical interventions, particularly upper GI surgeries, and also MBS due to unintended diaphragm damage with energy devices [2].

CT scan of the chest is the gold standard for diagnosis because the early symptoms may resemble the chronic pain and colic that usually accompany gastric bypass surgery, or even nonspecific symptoms like dyspnea and shortness of breath [3].

Management of these types of hernia is essentially surgical, preceded by resuscitation, because they usually remain silent till presenting with intestinal obstruction [4].

Iatrogenic diaphragmatic hernias following bariatric surgeries are exceedingly rare, with only three case reports documented in the literature and no large-scale studies conducted to date, to the best of our knowledge [5–7].

## Case Presentation

We present the case of a 36-year-old female with an unremarkable medical history who underwent Roux-en-Y gastric bypass (RYGB) 9 years prior to presentation for the management of morbid obesity. She achieved a good clinical response, losing almost 90% of her excess weight.

Six years after the bypass, she began to experience episodes of reflux and weight regain. Following investigations, a gastro-gastric fistula was diagnosed and successfully dismantled laparoscopically.

One year later, the patient began to report recurrent episodes of vague abdominal pain. Investigations revealed calculous gallbladder disease. The pain was attributed to gallstones and to chronic pain that may occur after RYGB. The patient presented acutely with sudden, severe abdominal pain that radiated to the back; upon examination, she was found to be tachycardic, normotensive, and afebrile, with abdominal tenderness mainly in the left hypochondrium.

She was admitted to the hospital, resuscitated, and investigations were conducted.

**Fig. 1:**
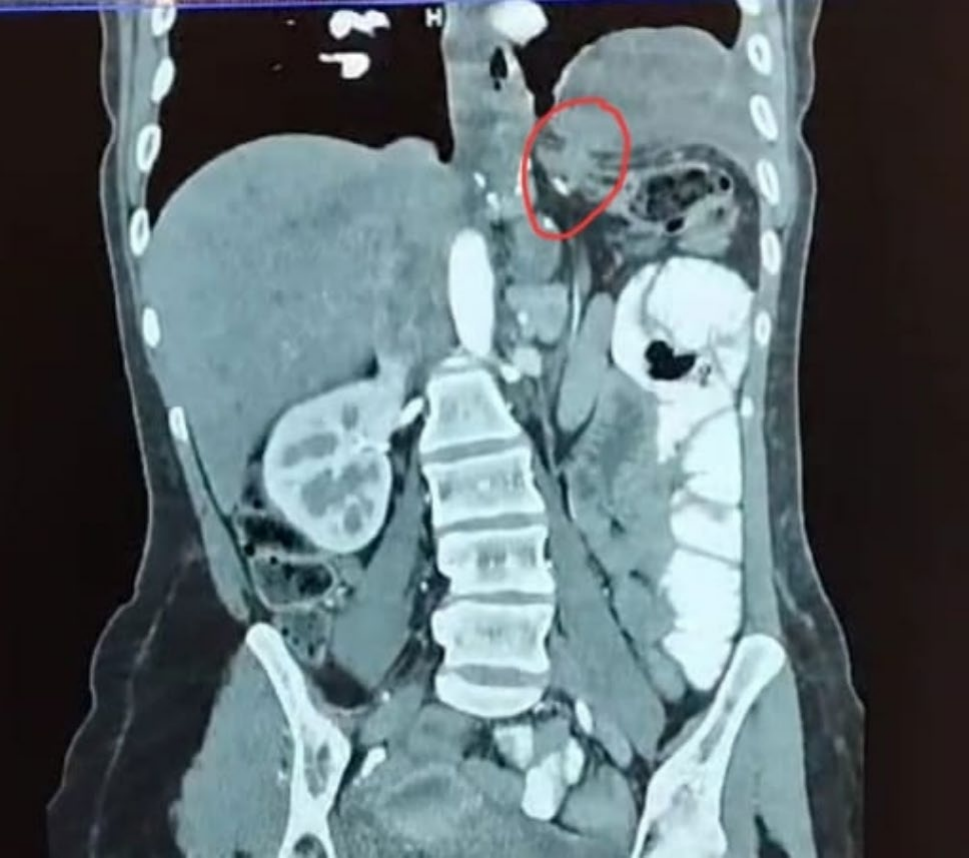
CT chest and abdomen showing herniation of a hollow organ through a small diaphragmatic opening

Laboratory tests revealed no significant findings apart from mild anemia and an elevated C-reactive protein (CRP) of 38 mg/L. A computed tomography (CT) scan of the abdomen and pelvis showed a hollow organ herniating into the left hemithorax, compressing the left lung with no intra-abdominal or thoracic collections (Fig. 1). After counseling the patient, the decision was made to proceed with laparoscopic exploration.

**Fig. 2:**
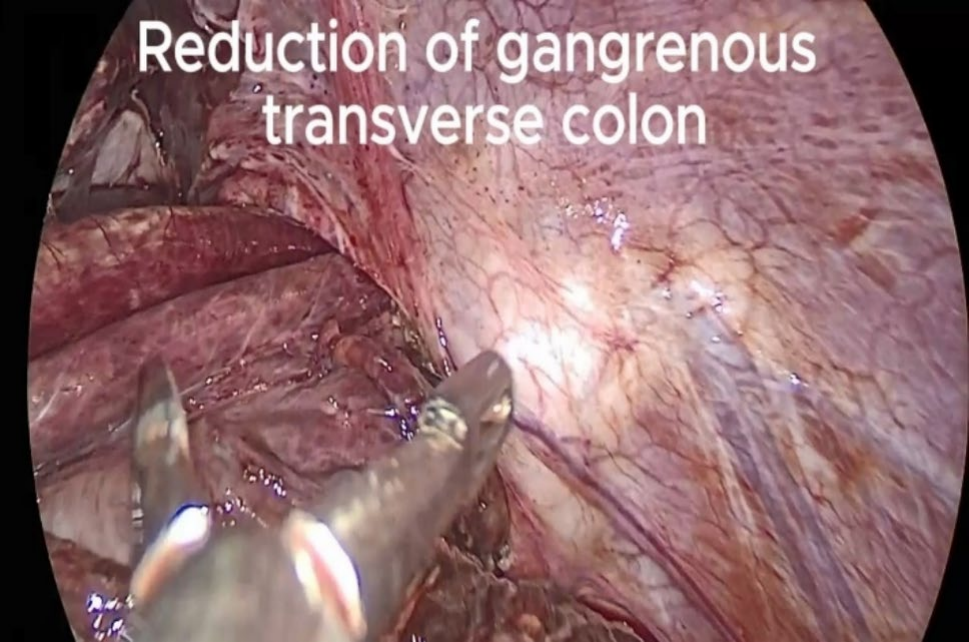
Reduction of the gangrenous colon from the diaphragmatic tear

We started the procedure with careful adhesiolysis. At this time, part of the transverse colon was found to be herniating through a diaphragmatic defect (Fig. 2) in the left posterior hemidiaphragm near the hiatal opening. This most likely resulted from inadvertent injury of the diaphragm by an ultrasonic shear on monopolar electrocautery during the second surgery that passed unnoticed.

The colon was edematous, and retraction was challenging, so the diaphragmatic opening was widened to facilitate its reduction.

**Fig. 3:**
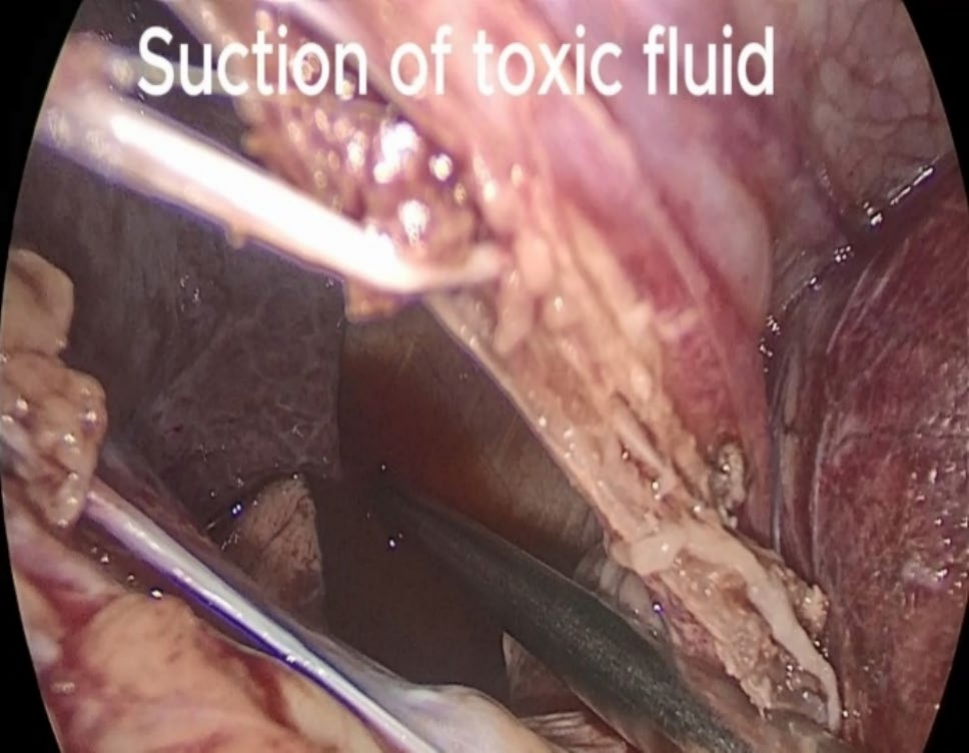
Suction and lavage of the left hemithorax

Upon exploring the left hemithorax, the left lung was collapsed, and toxic fluid was in the left hemithorax. (Fig. 3). We began by suctioning and lavaging the toxic fluid, followed by selective cannulation of the left lung by the anesthetist until it was completely inflated.

Resection of the strangulated segment of the transverse colon was performed, followed by creating an isoperistaltic colo-colic anastomosis using endo-staplers.

**Fig. 4:**
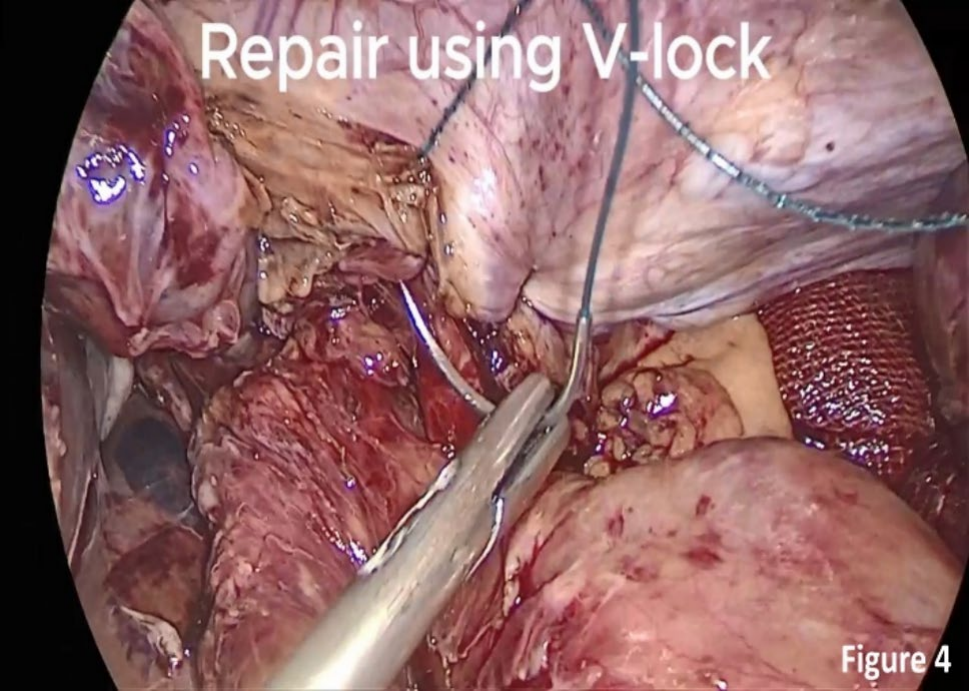
Closure of the diaphragmatic tear

The defect was closed by running sutures with V-Loc™ 180 Absorbable Sutures (Medtronic, Minneapolis, MN, USA) and reinforced with non-absorbable Polypropylene sutures. A concomitant hiatus hernia was detected and repaired with a cruroplasty and esophagopexy using Ethibond™ (Ethicon, Somerville, NJ, USA) (Fig. 4).

Postoperatively, she had a smooth recovery, and she was discharged home after 4 days of admission, until the fourth postoperative week, when she began to complain of difficulty breathing and left-sided pleuritic chest pain. Examination revealed limited air entry to the left lung, with no signs of sepsis or vital instability.

CT chest revealed left thoracic effusion completely occluding the left lung; upon consulting cardiothoracic surgeons, they decided to go for tapping instead of inserting a chest drain. Tapping was done twice, followed by a remarkable improvement of chest condition and complete inflation of the left lung in follow-up imaging. She subsequently experienced an uneventful postoperative recovery, as documented in follow-up visits.

## Discussion

This case report highlights a rare but crucial topic of iatrogenic diaphragmatic hernia. While the incidence of diaphragmatic hernias is low, those following bariatric surgeries are exceedingly rare.

This case underlines the importance of early diagnosis and proper surgical intervention, which can avoid unnecessary morbidities and mortality.

In this case, the patient had chronic vague symptoms of epigastric pain, but what grabbed attention was the sudden onset of severe abdominal pain with tachycardia, raising suspicion of a more serious underlying condition. The CT scan confirmed the diagnosis of the herniated colon in the left hemithorax.

Intraoperatively, finding a herniating, strangulated colon was consistent with the preoperative clinical and radiological findings.

As well known, diaphragmatic tears should be closed with non-absorbable sutures. Still, due to the lack of availability of non-absorbable barbed sutures and difficulty in closing the defect with braided sutures due to the dynamic nature of the diaphragm, we used absorbable barbed suture first, which was reinforced with non-absorbable propylene sutures; both were running continuous sutures.

In our belief, this will not be different from any diagrammatic hernia resulting from trauma or any other type of surgery.

## Postoperative Pleural Effusion: Management Considerations

The patient developed left-sided pleural effusion in week 4 postoperatively. This could be due to an inflammatory response following diaphragmatic repair and thoracic lavage, or it might be reactive hydrothorax from lung re-expansion. After consultation with the cardiothoracic team, they decided to go for tapping instead of inserting a chest drain. According to them, there was no active pathology to allow the chest to recollect, and there was no evidence of infection or hemodynamic instability. They tapped twice, achieving a successful outcome. This approach prevented complications like tube displacement or subcutaneous emphysema.

The probable etiology of the diaphragmatic tear is mostly unrecognized thermal injury from previous surgeries. This aligns with literature stating that energy devices such as electrocautery and ultrasonic coagulation shears are implicated in inadvertently injuring the diaphragm intraoperatively. The interval between primary surgery and hernia development varies [8].

Surgical repair is necessary for symptomatic diaphragmatic hernia, but the indications for asymptomatic diaphragmatic hernia remain unclear. Significant morbidity and even mortality are associated with delayed diagnosis due to the rarity of this condition [9].

After a thorough search, we found that only nine cases of iatrogenic diaphragmatic hernias after bariatric surgeries are reported in the literature, which are listed in Table 1 [10–18]. All the reported cases were either after Roux-en-Y Gastric Bypass (RYGB) or adjustable gastric band.

**Table 1:** Nine cases of iatrogenic diaphragmatic hernias after bariatric surgeries are reported in the literature

Author’s name	Journal	Year of publication	Initial procedure
Garcia et al.	Cureus	2022	RYGB
Abu-Jaish et al.	Bariatric Times	2016	RYGB
Alfa-Wali et al.	International Journal of Surgery	2013	RYGB
Borg et al.	Surgery for Obesity and Related Diseases	2011	RYGB
Arsalane et al.	Obesity Surgery	2005	RYGB
Dukhno et al.	International Surgery Journal	2006	Band
Boyce et al.	Journal of Surgical Case Reports	2008	Band
Batumsky et al.	Obesity Surgery	2015	Band
Fraga et al.	International Surgery Journal	2020	Band

## Limitations

- ⚬ A single case presentation cannot be generalized.
- ⚬ Short-term follow-up.

## Conclusion

Iatrogenic diaphragmatic hernia is a rare and potentially fatal condition. Diagnosis can be challenging due to the vague presentation, which may be attributed to more common conditions. Early diagnosis and proper surgical intervention can help to avoid morbidities, if not mortality.

Careful handling of energy devices near the diaphragm, along with awareness of the potential for iatrogenic diaphragmatic hernia after bariatric surgeries, is necessary to avoid this potentially fatal condition.

## Supplementary Information

Below is the link to the electronic supplementary material.

Supplementary file1 (MOV 324182 KB)

## Data Availability

No datasets were generated or analyzed during the current study.
